# A three-dimensional kinematic analysis of bipedal walking in a white-handed gibbon (*Hylobates lar*) on a horizontal pole and flat surface

**DOI:** 10.1007/s10329-024-01177-x

**Published:** 2025-01-15

**Authors:** Takafumi Fujiwara, Kohta Ito, Tetsuya Shitara, Yoshihiko Nakano

**Affiliations:** 1 Laboratory of Biological Anthropology, Graduate School of Human Sciences, Osaka University, Suita, Osaka Japan; 2 Artificial Intelligence Research Center, National Institute of Advanced Industrial Science and Technology (AIST), Koto-Ku, Tokyo, Japan

**Keywords:** Gibbon, Bipedal walking, Pole and flat condition, Joint angle, Step width

## Abstract

Gibbons, a type of lesser ape, are brachiators but also walk bipedally and without forelimb assistance, not only on the ground but also on tree branches. The arboreal bipedal walking strategy of the gibbons has been studied in previous studies in relation to two-dimensional (2D) kinematic analysis. However, because tree branches and the ground differ greatly in width, leading to a constrained foot contact point on the tree branches, gibbons must adjust their 3D joint motions of trunk and hindlimb on the tree branches. Furthermore, these motor adjustments could help minimize the center of mass (CoM) mediolateral displacement. This study investigated the kinematic adjustment mechanism necessary to enable a gibbon to walk bipedally on an arboreal-like substrate using 3D measurements. Trials were recorded with eight video cameras that were placed around the substrate. The CoM position on the body, the Cardan angles of the hindlimb joints and trunk, and spatiotemporal parameters were calculated. Asymmetry of thorax, pelvis, trunk, and left and right hindlimb joint motion was observed in the pole and flat conditions. In the pole condition, the narrower step width and the smaller range of motion of the mediolateral CoM displacement were observed with increased hip adduction and knee eversion angles. These kinematic adjustments might place the knee and foot directly under the body during the single support phase, producing a reduced step width and the amount of the mediolateral CoM displacement of a gibbon.

**Supplementary Information:**

The online version contains supplementary material available at 10.1007/s10329-024-01177-x.

## Introduction

Gibbons, lesser apes inhabiting the forest canopy of the tropical rainforests of Southeast Asia, use a wide range of posturo-locomotor modes (Baldwin and Teleki 1976; Fan et al. 2013; Fleagle 1976, 2013; Gitten 1983; Nowak and Reichard 2016). Their primary mode is brachiation (Fleagle 2013; Gitten 1983), although bipedalism is also included in their repertoire (Cannon and Leighton 1994; Fan et al. 2013; Fleagle 1976; Vereecke et al. 2006a, b). In captivity, gibbons exhibit the highest incidence of bipedal walking among apes (Rosen et al. 2022) and show a certain reliance on bipedalism in their locomotor behavior.

Gibbons’ bipedalism has been examined from the kinematic (Hirasaki and Kumakura 2004; Ishida et al. 1984; Kinoshita et al. 2022; Okada 1985; Schmid and Piaget 1994; Vereecke et al. 2006b), kinetic (Kimura 1985; Kimura et al. 1977; Vereecke and Aerts 2008; Vereecke et al. 2006c), and electromyographic (Goto et al. 2023; Ishida et al. 1985; Kumakura 1989; Okada and Kondo 1982; Shapiro and Jungers 1988; Stern and Susman 1981, 1983) perspectives. Being profoundly arboreal primates (Fleagle 1976, 2013; Gitten 1983; Tanaka 2024), gibbons exhibit bipedalism not only on the ground but also on tree branches (Vereecke et al. 2006a; Yamazaki and Ishida 1984). Previous studies have provided many insights into gibbons’ bipedal walking on flat surfaces, but a comprehensive understanding of the nature of the bipedalism in the gibbons is lacking. To understand the locomotor behavior of gibbons in the wild environment, it is necessary to examine their bipedal walking on tree branches rather than on flat surfaces.

The majority of previous investigations of gibbons’ bipedalism have focused on observing it on flat surfaces, although some attention has also been directed toward bipedalism on tree branches (Tanaka 2024; Vereecke et al. 2006a; Yamazaki and Ishida 1984). For example, Yamazaki and Ishida (1984) compared the kinematics of the bipedal walking of gibbons between on the ground and on a pole and found that gibbons flexed their hip and knee joints to a greater extent on the pole compared than on the ground. Likewise, Vereecke et al. (2006a) found that the stride length, average velocity, and duty factor were greater on branches than on a flat catwalk. They suggest that the changes in these parameters are characteristic of compliant gait, which is adaptive to arboreal locomotion and might have contributed to improved balance and reduced tree branch vibration (Vereecke et al. 2006a). These studies identified locomotor adaptations in gibbons’ bipedalism on branches using 2D kinematic analysis, locating the kinematic alteration in the sagittal plane. However, such studies may not fully clarify gibbons’ bipedal walking on branches. The width of the substrate is narrower on branches than on the ground, and the foot contact point is necessarily restricted. Due to foot placement, it is predicted that gibbons would require 3D kinematic adjustment in the trunk and hindlimb joints on a pole substrate to minimize the mediolateral movement of the body CoM, thus reducing the risk of falling. In quadrupedal primates, for instance, the feet are positioned beneath the trunk with slightly abducted hip and adducted knee on a pole substrate (Carlson and Demes 2010; Schmidt 2005). However, it remains unclear how gibbon adjusts their whole-body 3D kinematics in response to difference in the substrate conditions between pole and the flat surface.

To identify gibbon ability to arboreal bipedal walking, it is necessary to investigate the detailed kinematic characteristics of pole walking, not only in the sagittal plane but also in the frontal and horizontal planes. This study examined the locomotor characteristics of gibbons enabling them to walk on a pole substrate relative to walking on the ground using 3D kinematic measurements.

## Materials and methods

### Experimental subject and protocol

The animal used in the experiment was a white-handed gibbon (*Hylobates lar*, female, 27 years old, body weight 7.12 kg, lower leg length = 0.16 m, calculated as the mean 3D distance from the tibial lateral condyle to the tip of the calcaneal ridge over the stride cycle (Aerts et al. 2000)). All experimental protocols were approved by the Institutional Animal Care and Use Committee of the Graduate School of Human Sciences of Osaka University (R2-1–0). White-handed gibbons are distributed across Southeast Asia and live the forest canopy (Fleagle 2013). While gibbons are not unique in exhibiting bipedal walking (Nowak and Reichard 2016), they tend to use this locomotor mode more frequently than other apes (Rosen et al. 2022).

### Landmarks and body segments

**Fig. 1:**
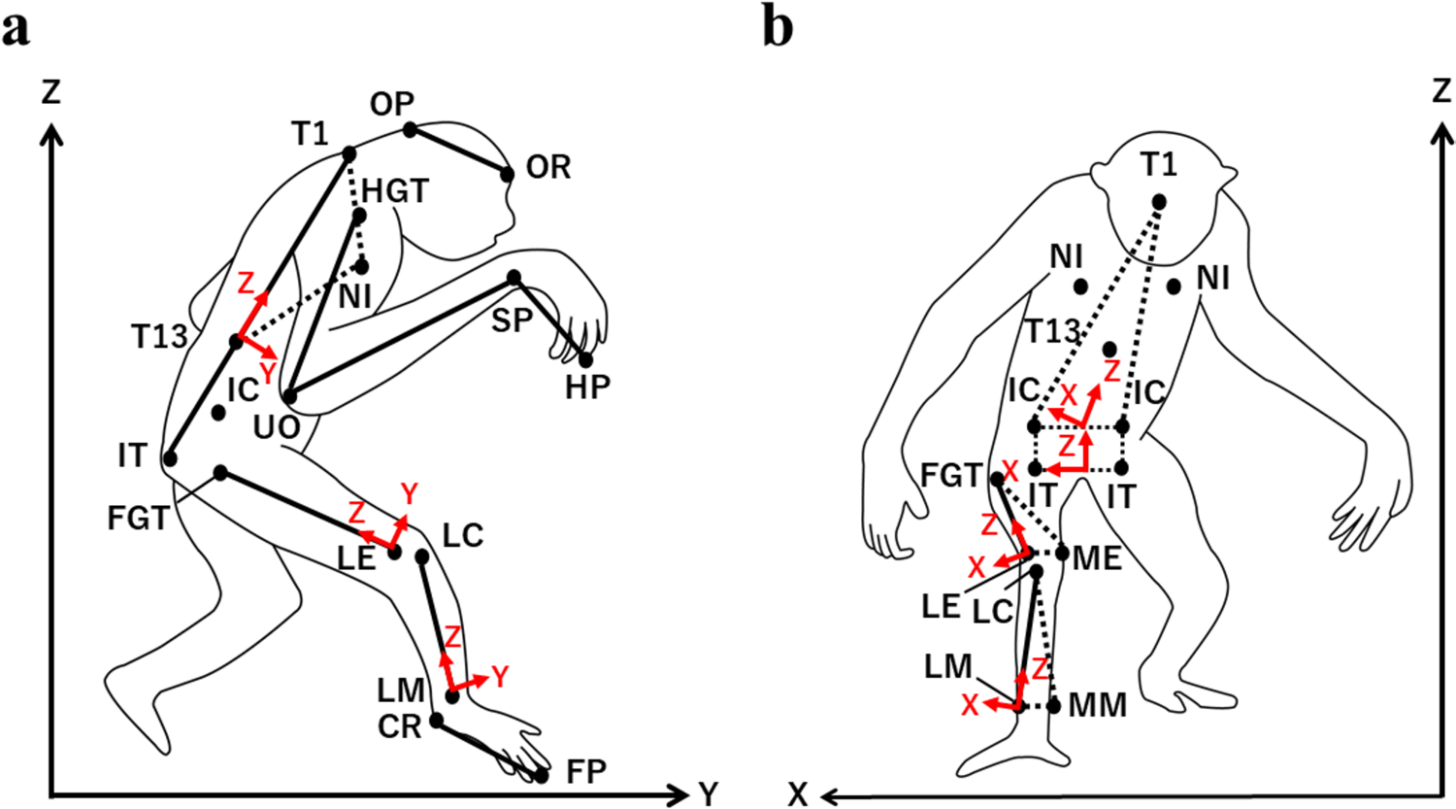
Landmarks set in a global coordinate system in the sagittal (**a**) and frontal (**b**) view. The landmarks are denoted only on the right side here, but were marked on both sides in experiments. Red arrows represent the local coordinate systems that align with the cardinal anatomical axes: mediolateral (x), anteroposterior (y), and superoinferior (z). **a** OP: occipital protuberance, OR: orbital ridge, T1: first thoracic spinous process, T13: thirteenth thoracic spinous process, NI: nipple, IT: ischial tuberosity, IC: the most cranial points of iliac crest, HGT: humeral greater tubercle, UO: ulnar olecranon process, SP: ulnar styloid process, HP: the tip of third hand phalange, FGT: femoral greater trochanter, LE: femoral lateral epicondyle, LC: tibial lateral condyle, LM: lateral malleolus, CR: the tip of calcaneal ridge, and FP: third foot phalange. **b** ME: femoral medial epicondyle and MM: medial malleolus

To calculate the position of the CoM of the gibbon’s body, we constructed a model of it. The body was divided into 14 segments: head, trunk, and left and right upper arms, forearms, hands, thighs, shanks, and feet, using 27 landmarks: occipital protuberance, orbital ridge, first thoracic spinous process, and left and right ischial tuberosities, the most cranial points of iliac crest, humeral greater tubercles, ulnar olecranon processes, ulnar styloid processes, the tips of third hand phalange, femoral greater trochanters, femoral lateral epicondyles, tibial lateral condyles, lateral malleoluses, calcaneal ridge, the tip of third foot phalanges (Fig. 1; based on Crompton et al. 1996). For the thorax and hindlimbs, thirteenth thoracic spinous process, left and right nipples, and medial landmarks (the femoral medial epicondyle and medial malleolus) were marked to calculate the Cardan angles (Shitara et al. 2022; Thompson et al. 2018). The mass and positional vector for each segment were calculated using the percentage of the mass of each segment relative to the body mass and the segmental CoM position from the proximal point, respectively (Isler et al. 2006; Table 1). Specifically, the mass percentages of each segment were calculated as follows. First, based on Tables 1, 2, 3 of Isler et al. (2006), the mass of the trunk segment was recalculated by subtracting the total mass of the non-trunk segments from the estimated body weight. Since the mass of the trunk segment reported in Table 3 of Isler et al. (2006) was measured after evisceration, it was necessary to estimate the value including the viscera for application to the gibbon used in this study. Next, the mass of each body segment for three gibbons (Hy1-Hy3 in Isler et al. (2006)) was calculated as a percentage of their estimated body weight by dividing the segment mass by the body weight and multiplying by 100. These percentages were then averaged across individuals to obtain the mean segmental mass percentage, which was used to estimate the segmental masses of the gibbon in this study (Table 1). The CoM position percentages were averaged the CoM positions within each segment of Hy1-3 as reported in Table 3 of Isler et al. (2006) (see Supplementary Information for the body weight and mass of each segment and CoM position percentages of Hy1-3 in Isler et al. (2006)).

**Table 1:** Segmental CoM position and mass percentages of gibbon’s body, defined as the mean of three gibbons in the previous study (upper and middle row, Isler et al. 2006), and segmental mass of the gibbon’s body used in the current study (lower row, calculated)

	Head	Trunk	Upper arm	Forearm	Hand	Thigh	Shank	Foot
CoM position percentage [%]	44.5	46.9	48.0	44.4	50.6	45.9	46.9	48.5
Mass percentage [%]	7.20	65.90	3.40	2.60	1.00	3.70	1.70	1.00
Mass of segment [kg]	0.51	4.69	0.24	0.19	0.07	0.26	0.12	0.07

Note that the mass of the fore- and hindlimbs are only those of one side

**Table 2:** Comparison of the spatiotemporal parameters between the pole and flat conditions in the left and right strides

Abbreviation	Parameter	Formula^a^	This study (right stride)		*p*	This study (left stride)		*p*	Vereecke et al. (2006a)	
			Flat	Pole		Flat	Pole		Flat	Pole
CT	Contact time [s]		$0.34\pm 0.02$	$0.33\pm 0.04$	0.19	$0.35\pm 0.02$	$0.34\pm 0.04$	0.26	$0.43\pm 0.12$	$0.46\pm 0.11$
SC	Stride cycle time [s]		$0.52\pm 0.03$	$0.51\pm 0.04$	0.62	$0.53\pm 0.03$	$0.53\pm 0.04$	0.62	–	–
CH	Swing phase time of contralateral hindlimb [s]		$0.26\pm 0.02$	$0.27\pm 0.02$	0.75	$0.27\pm 0.02$	$0.27\pm 0.02$	0.92		
SS	Stride symmetry	CH/SC	$0.49\pm 0.03$	$0.51\pm 0.01$	0.25	$0.50\pm 0.02$	$0.49\pm 0.02$	0.69		
SL	Stride length [m]		$0.53\pm 0.04$	$0.56\pm 0.05$	0.45	$0.57\pm 0.02$	$0.60\pm 0.06$	0.25	$0.88\pm 0.19$	$0.97\pm 0.21$
DSL	Dimensionless stride length	SL/LL	$3.34\pm 0.23$	$3.49\pm 0.32$	0.36	$3.53\pm 0.11$	$3.78\pm 0.35$	0.10	$5.29\pm 1.18$	$6.25\pm 1.64$
V	Average velocity [m/s]	SL/SC	$1.03\pm 0.10$	$1.10\pm 0.17$	0.50	$1.08\pm 0.07$	$1.13\pm 0.16$	0.34	$1.36\pm 0.43$	$1.44\pm 0.52$
DV	Dimensionless velocity	V/(LL $\times$ g)^1/2^	$0.83\pm 0.08$	$0.87\pm 0.14$	0.45	$0.86\pm 0.05$	$0.91\pm 0.13$	0.29	$1.12\pm 0.38$	$1.18\pm 0.44$
RSL	Right step length [m]		$0.33\pm 0.03$	$0.30\pm 0.03$	0.08	$0.32\pm 0.02$	$0.32\pm 0.04$	0.92		
LSL	Left step length [m]		$0.20\pm 0.04$	$0.25\pm 0.02$	*	$0.25\pm 0.01$	$0.27\pm 0.02$	*		
DF	Duty factor	CT/SC	$0.69\pm 0.02$	$0.66\pm 0.03$	0.11	$0.68\pm 0.02$	$0.64\pm 0.03$	*	$0.63\pm 0.06$	$0.65\pm 0.06$
SW	Step width [m]		$0.09\pm 0.01$	$0.03\pm 0.01$	**	$0.09\pm 0.01$	$0.03\pm 0.01$	**	–	–
DSW	Dimensionless step width	SW/LL	$0.56\pm 0.07$	$0.17\pm 0.08$	**	$0.56\pm 0.06$	$0.17\pm 0.07$	**	–	–
SF	Stride frequency [Hz]	1/SC	$1.94\pm 0.11$	$1.97\pm 0.16$	0.62	$1.89\pm 0.11$	$1.89\pm 0.14$	0.62	$1.54\pm 0.27$	$1.49\pm 0.34$
DSF	Dimensionless stride frequency	SF$\times$(LL/g)^1/2^	$0.25\pm 0.01$	$0.25\pm 0.02$	0.82	$0.24\pm 0.01$	$0.24\pm 0.02$	0.77	$0.21\pm 0.04$	$0.18\pm 0.03$

*p < 0.05, **p < 0.01

^a^*LL* lower leg length [m]; *g* he gravitational acceleration = 9.81 m/s^2^

**Table 3:** Comparison of the center of mass (CoM) height and joint angles at the initial contact (IC), midstance (Mst), and terminal stance (Tst) between the pole and flat conditions

Parameter	IC			Mst			Tst		
	Flat	Pole	*p*	Flat	Pole	*p*	Flat	Pole	*p*
CoM height [cm]	$35.7\pm 1.4$	$33.9\pm 1.0$	*	$34.7\pm 0.9$	$33.2\pm 0.7$	**	–	–	–
Trunk extension (right) [deg]	$-31.9\pm 3.5$	$-36.7\pm 3.8$	*	$-39.6\pm 3.4$	$-42.6\pm 4.3$	0.06	$-36.7\pm 3.7$	$-40.2\pm 4.5$	0.05
Trunk extension (left) [deg]	$-38.8\pm 2.1$	$-40.0\pm 4.2$	0.77	$-39.5\pm 1.7$	$-41.9\pm 3.7$	0.18	$-38.4\pm 1.7$	$-40.6\pm 4.3$	0.34
Trunk lateral flexion (right) [deg]	$-15.2\pm 6.6$	$-12.2\pm 2.5$	0.17	$-7.0\pm 6.4$	$-6.1\pm 3.3$	0.55	$-17.0\pm 7.6$	$-11.6\pm 2.8$	0.11
Trunk lateral flexion (left) [deg]	$-6.8\pm 6.9$	$-5.9\pm 4.7$	0.92	$-15.5\pm 7.2$	$-13.5\pm 3.1$	0.63	$-7.8\pm 7.0$	$-6.9\pm 1.6$	0.63
Trunk axial rotation (right) [deg]	$26.3\pm 8.0$	$26.6\pm 7.1$	0.76	$16.9\pm 8.3$	$17.3\pm 5.8$	0.88	$9.8\pm 8.9$	$-2.0\pm 5.2$	**
Trunk axial rotation (left) [deg]	$-0.7\pm 9.2$	$-2.2\pm 7.8$	0.77	$11.2\pm 8.4$	$7.9\pm 7.1$	0.45	$20.0\pm 7.9$	$26.1\pm 6.3$	0.15
Thorax extension (right) [deg]	$-37.5\pm 2.9$	$-42.9\pm 3.8$	**	$-45.6\pm 3.5$	$-48.9\pm 4.3$	*	$-43.4\pm 3.2$	$-47.3\pm 4.4$	*
Thorax extension (left) [deg]	$-45.4\pm 2.4$	$-46.1\pm 4.1$	0.63	$-45.9\pm 2.1$	$-48.5\pm 3.9$	0.08	$-44.4\pm 2.1$	$-47.0\pm 4.4$	0.25
Thorax lateral flexion (right) [deg]	$-17.2\pm 7.1$	$-14.0\pm 2.7$	0.17	$-8.8\pm 7.2$	$-7.7\pm 3.7$	0.50	$-18.6\pm 8.6$	$-11.9\pm 3.5$	0.11
Thorax lateral flexion (left) [deg]	$-8.7\pm 8.0$	$-7.3\pm 6.2$	0.77	$-18.0\pm 8.7$	$-15.1\pm 4.2$	0.56	$-11.0\pm 8.2$	$-9.8\pm 2.7$	0.56
Thorax axial rotation (right) [deg]	$10.3\pm 8.1$	$19.4\pm 6.8$	*	$5.8\pm 6.6$	$11.5\pm 4.9$	*	$-0.4\pm 6.4$	$-2.4\pm 4.5$	0.26
Thorax axial rotation (left) [deg]	$-0.1\pm 8.3$	$0.1\pm 6.0$	0.85	$6.9\pm 8.4$	$5.9\pm 5.5$	0.56	$16.5\pm 7.6$	$21.9\pm 6.6$	0.15
Pelvis tilt (right) [deg]	$-9.8\pm 2.6$	$-12.2\pm 1.9$	*	$-10.0\pm 2.1$	$-12.2\pm 2.3$	*	$-11.6\pm 0.8$	$-15.5\pm 1.5$	**
Pelvis tilt (left) [deg]	$-14.1\pm 3.3$	$-16.3\pm 3.9$	0.25	$-10.7\pm 1.6$	$-12.9\pm 2.5$	0.08	$-10.3\pm 1.1$	$-13.0\pm 1.2$	**
Pelvis obliquity (right) [deg]	$-3.5\pm 1.7$	$-2.3\pm 1.1$	0.11	$0.5\pm 1.9$	$4.5\pm 1.6$	**	$-4.8\pm 2.6$	$-1.4\pm 2.3$	**
Pelvis obliquity (left) [deg]	$2.1\pm 2.6$	$2.6\pm 3.0$	0.39	$-3.1\pm 1.9$	$-3.4\pm 1.6$	0.56	$1.3\pm 3.0$	$2.9\pm 1.6$	0.29
Pelvis axial rotation (right) [deg]	$34.2\pm 11.5$	$38.9\pm 10.0$	0.23	$12.8\pm 12.6$	$15.7\pm 6.3$	0.71	$2.2\pm 12.2$	$-6.4\pm 4.9$	0.08
Pelvis axial rotation (left) [deg]	$-6.5\pm 14.4$	$-10.6\pm 6.5$	0.63	$15.9\pm 12.7$	$14.3\pm 7.5$	0.70	$28.9\pm 12.1$	$32.4\pm 6.9$	0.44
Hip flexion (right) [deg]	$73.3\pm 3.7$	$79.3\pm 2.6$	**	$68.1\pm 2.3$	$71.0\pm 3.6$	0.08	$58.0\pm 3.4$	$54.6\pm 3.8$	0.07
Hip flexion (left) [deg]	$80.8\pm 2.1$	$85.3\pm 4.3$	*	$66.7\pm 2.9$	$70.7\pm 4.3$	0.05	$55.0\pm 5.3$	$54.7\pm 6.5$	0.92
Hip adduction (right) [deg]	$-26.3\pm 8.1$	$-19.8\pm 10.1$	0.17	$-11.8\pm 5.5$	$-5.5\pm 6.0$	*	$2.1\pm 4.7$	$6.9\pm 4.9$	0.10
Hip adduction (left) [deg]	$-14.3\pm 9.0$	$-10.1\pm 4.2$	0.05	$-13.4\pm 6.9$	$-5.6\pm 5.3$	*	$-7.1\pm 5.5$	$-0.3\pm 5.4$	*
Knee extension (right) [deg]	$-59.5\pm 4.1$	$-59.2\pm 6.5$	0.94	$-100.7\pm 3.3$	$-104.5\pm 3.9$	*	$-128.7\pm 5.9$	$-121.8\pm 4.9$	*
Knee extension (left) [deg]	$-62.3\pm 2.5$	$-61.4\pm 3.6$	0.25	$-96.8\pm 2.5$	$-101.5\pm 1.5$	**	$-126.8\pm 5.4$	$-122.4\pm 6.3$	0.17
Knee inversion (right) [deg]	$-2.4\pm 10.0$	$-11.0\pm 10.7$	0.11	$0.7\pm 5.7$	$-6.7\pm 4.4$	*	$-0.3\pm 15.6$	$-3.3\pm 15.3$	0.71
Knee inversion (left) [deg]	$3.6\pm 5.3$	$1.1\pm 4.6$	0.29	$8.8\pm 3.4$	$3.8\pm 3.4$	**	$9.0\pm 14.8$	$2.7\pm 14.9$	0.34

*p < 0.05, **p < 0.01

### Local coordinate systems

The local coordinate system was defined on the trunk, thorax, pelvis, thigh, and shank segments. We defined the local coordinate systems by calculation on the xyz axes. These axes corresponded to the mediolateral, anteroposterior, and superoinferior axes of the given bone, respectively (Fig. 1) (see Supplementary Information for the detailed explanation of the local coordinate system definition). The reference posture was as the fully extended posture (as in the human anatomical posture) with an orientation that matched the global coordinate system (Shitara et al. 2022) rather than as a specific posture taken during a stance phase (e.g., midstance).

### Data acquisition

**Fig. 2:**
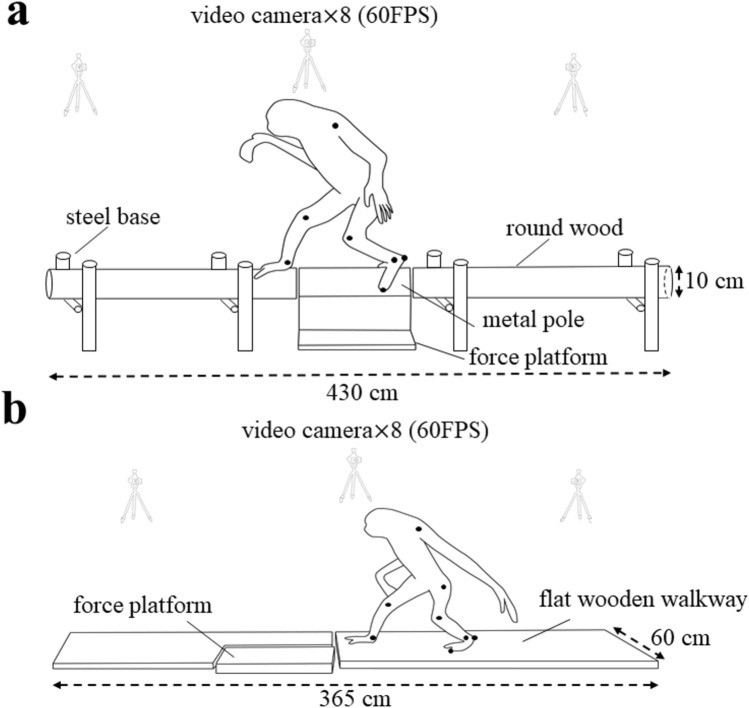
Experimental setup of the pole (**a**) and flat (**b**) condition. Bipedal walking on the flat and pole substrates were recorded using eight cameras placed around the substrates. The substrates were placed so that their long axis coincided with the direction of travel

Before the measurements, eight digital video cameras (NXCAM, SONY, Ltd) were placed around the substrate and calibrated using a calibration pole having six control points, stood vertically at every corner of the calibration space (in a static calibration). The calibration space was 100 cm wide, 150 cm long, and 110 cm high. The error of the control points was 0.38 ± 0.06 cm. After calibration, the animal’s hair was removed and landmarks were marked with nontoxic markers under gas sevoflurane anesthesia that lasted for approximately 1 h. About 30 min later, the animal awoke, and the trials were filmed by cameras at 60 FPS with 1920 $\times$ 1080 pixel (Fig. 2). Two substrate conditions were included: a flat substrate condition (flat condition; 60 cm wide, 365 cm long, and 9.5 cm high from the floor to top surface of the substrate) and a pole substrate condition (pole condition; 10 cm in diameter, 430 cm total length, and 25.6 cm high, from the floor to the top of the pole). A force platform was settled in the middle of the walkway to measure the preliminary force data for use in another study. The experimenter guided the animal’s direction of travel using a leash.

The video data for each trial (beginning with the contact of the right foot and end with the next contact of the same foot) were obtained using video editing software (EDIUS Pro 9; Grass Valey Co., Ltd). Ten trials were examined for each condition, based on the following criteria: trials where the subject walked at least 11 steps (for the pole condition) or 8 steps (for the flat condition) from one end of the substrate to the other, without stopping and descending from the substrate. Since the gait of the subject was bilaterally asymmetrical, the left stride was also analyzed (nine trials in the pole condition and eight trials in the flat condition, for each). In the pole condition, the initial contact of the left foot occurred one step prior to the initial contact of the right foot during the right stride, whereas in the flat condition, the stride occurred one step later. The left stride was defined as one stride before the right stride in the pole condition but as one stride after the right stride in the flat condition. This distinction was made because, in the pole condition, the force plate was positioned closer to the end of the walkway, which could potentially cause deceleration if the stride after the right stride were used, as in the flat condition.

**Fig. 3:**
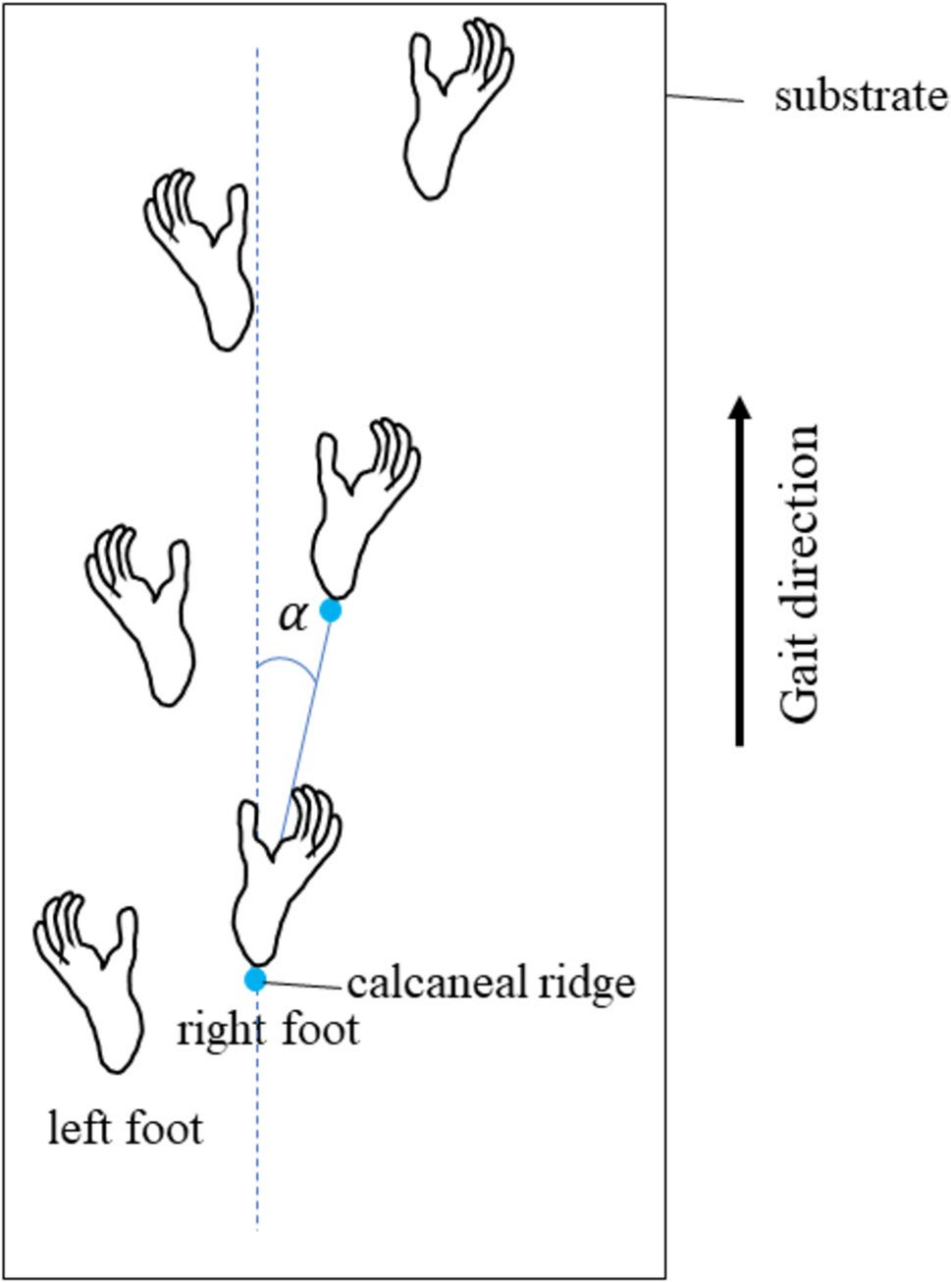
Schematic image of the foot contact positions in the horizontal plane in the flat condition showing right stride cycle. To control the direction of walking in the flat condition, the coordinates of each landmark in all trials were transformed *α* degrees between the line segment that connected the right calcaneal ridge at the initial contact and at the next initial contact (solid blue line), and a line parallel to the substrate (broken blue line) in the horizontal plane

The landmarks were digitized frame-by-frame, using a 3D kinematic analysis software (Frame Dias V; Q’sfix Co., Ltd.). The raw coordinate data were smoothed using a 4th Butterworth low-pass filter having a cutoff frequency of 7 Hz (Vereecke et al. 2006b). The length of the trial data was normalized to 101 frames. In the pole condition, the coordinates of the landmarks were transformed using the inclination angle of the substrate in the horizontal plane. In the flat conditions, the gibbon walked diagonally in the direction of travel on most trials (Fig. 3), so each 3D coordinate value was transformed to control for walking direction. The coordinates of the landmarks were transformed by $\alpha$ degrees between the line segment connecting the right CR (or the left CR in the left stride) at initial contact and at next initial contact with a line parallel to the substrate, such that the values of the mediolateral component of the gibbon’s CR coincided at 0% and 100% of the stride cycle for each trial (Fig. 3). With the use of landmark coordinate data, the following parameters were calculated in R 4.2.2 (R Core Team 2022).

### Data processing

Cardan angles (Kadaba et al. 1990; Ogihara et al. 2010; O’Neill et al. 2015) of the thorax, pelvis and trunk segment in the global coordinate system, and the hip and knee joints (with three degrees of freedom for each) were calculated by solving the following matrix (Ogihara et al. 2010):

1 $$\mathbf{R}\left(t\right)={\left[{\mathbf{M}}_{\text{pro}}\left(t\right)\right]}^{-1}{\mathbf{M}}_{\text{dis}}\left(t\right)=\left[\begin{array}{ccc}c\theta c\psi & -c\theta s\psi & s\theta \\ s\phi s\theta c\psi +c\phi s\psi & -s\phi s\theta s\psi +c\phi c\psi & -s\phi c\theta \\ -c\phi s\theta c\psi +s\phi s\psi & c\phi s\theta s\psi +s\phi c\psi & c\phi c\theta \end{array}\right]$$

where $\mathbf{R}(t)$ is a rotation matrix having an $x$-$y$-$z$ rotational sequence at time =$t$ (i.e., where the rotational sequence passes through flexion–extension, adduction-abduction, and internal–external rotation, in that order). ${\mathbf{M}}_{\text{pro}}(t)$ and ${\mathbf{M}}_{\text{dis}}(t)$ are local coordinate systems that are fixed on the proximal and distal segments of the global coordinate system, respectively (Fig. 1). $\phi$, $\theta$, and $\psi$ are the rotational angles around the $x$, $y$, and $z$ axes, respectively. $c$ and $s$ represent the cosine and sine, respectively. The motions in the horizontal plane of the hip and knee joints were not analyzed in this study because the motions in the sagittal and frontal planes of the hip joint change significantly during bipedal walking (Johnson et al. 2022), and the motion of the knee joint is also important for both the sagittal and frontal planes (O’Neill et al. 2015). The thorax, pelvis, and trunk angles were calculated as the changes of the pelvis and trunk coordinate systems in the global coordinate system, respectively.

The CoM position of the body was calculated as follows (Winter 2009):

2 $$\mathbf{b}\left(t\right)=\sum_{i=1}^{14}\frac{{\mathbf{p}}_{i}\left(t\right)\bullet {m}_{i}}{M}$$

where $\mathbf{b}(t)$ describes the positional vector of the CoM of the body at time = $t$. ${\mathbf{p}}_{i}(t)$ and ${m}_{i}$ refer to the positional vectors of the CoM at time = $t$ and the mass of the $i$ th body segment, respectively. $M$ represents the mass of the body.

The CoM height was calculated as forming the inner product of the vector, from the right LM to the CoM of the body and the vertical axis in the sagittal plane, as follows:

3 $$h\left(t\right)={\mathbf{d}}_{\text{yz}}(t)\bullet \mathbf{z}$$

where $h\left(t\right)$ is the CoM height at time = $t$. ${\mathbf{d}}_{\text{yz}}(t)$ is the 2D vector from the right LM to the CoM of the body at time =$t$ in the sagittal plane. $\mathbf{z}$ is the vertical axis of the global coordinate system. From a mechanical point of view, the CoM height should be defined as from the center of pressure (CoP) to the CoM. Because the gibbon’s foot contacts with the side of the pole substrate in the pole condition (Fig. 2a), the CoP should be below the top of the pole. As the height of the CoP decreases, the height of the ankle joint also decreases, such that the vertical distance from the right LM to the CoM was defined as the CoM height in this study.

The CoM mediolateral displacement was calculated as the mediolateral displacement of the body CoM relative to a baseline, calculated as the mean value of the mediolateral component of the CoM of the body in the stride cycle, as follows:

4 $$l\left(t\right)={b}_{\text{x}}\left(t\right)-\overline{{b }_{\text{x}}\left(t\right)}$$

where $l\left(t\right)$ is the CoM mediolateral displacement at time = $t$. ${b}_{\text{x}}(t)$ is the mediolateral component of the CoM of the body at time = $t$. $\overline{{b }_{\text{x}}\left(t\right)}$ describes the mean value of the mediolateral component of the CoM of the body throughout the gait cycle. To understand the contribution of the linear motion of the thorax and pelvis to the body CoM, the mediolateral displacements of the thorax and pelvis were calculated as the relative motion to the mean value of stride cycle of the mediolateral components of the centroids of these segments (Thompson et al. 2018). Also, the mediolateral distances between the CoM and the calcaneal ridges were calculated to concern mediolateral balance strategy between conditions.

The following spatiotemporal parameters were also calculated: stride length (anteroposterior distance between the heel of the right foot (left foot in left stride) at 0% and 100% of stride cycle), step length (anteroposterior distance between the heel of the right (or left) initial contact and left (or right) initial contact), step width (mediolateral distance between the left and right heels (CRs) during the double stance phase), duty factor (stance duration divided by stride cycle time), average velocity (stride length divided by stride cycle time), stride frequency (inverse of stride cycle time), and stride symmetry (ratio of the time from the beginning of the stride cycle to the contact of the contralateral hindlimb accounted for stride cycle time). If the stride symmetry is between 0.45 and 0.55, the trial is temporally symmetry (Cartmill et al. 2002). To enable comparison with previous study (Vereecke et al. 2006a) and to confirm that the gibbon that we used was not an extraordinary individual, dimensionless velocity, stride length, and stride frequency were also calculated.

Because several variables were not normally distributed, a non-parametric Mann–Whitney *U* test was performed to compare the pole and flat conditions at a 5% significance level.

## Results

In the pole condition, the gait direction angle ($\alpha$ degrees) was 1.1° to the right in the horizontal plane. In the flat condition, it was 2.9° ± 2.7° to the right during the right stride and 2.8° ± 2.3° to the right during the left stride in the horizontal plane. These angles were used to adjust the coordinates of the landmarks in the measurement space accordingly.

### Spatiotemporal parameters

Average velocity can affect many parameters of gait (Higurashi and Kumakura 2021); however, average velocity was not different between conditions (Table 2; right: *p* = 0.50; left: *p* = 0.34), which suggests that the difference between the conditions in this study could be caused by differences in substrate shape, not by gait velocity. All of the results, except for duty factor in the left stride and step width, showed no significant difference between conditions (Table 2). Step width was significantly reduced in the pole condition relative to the flat condition in both strides (Table 2; *p* < 0.01). Stride symmetry was not significantly different between conditions in both strides and ranged from 0.45 to 0.55, indicating the gait was temporally symmetrical (Table 2). On the other hand, bilateral asymmetry was observed in the step length (Table 2), and the trunk and hindlimb joint angles in both strides (see below). Step length was larger on the right step than on the left step in both strides (Table 2).

### Trunk kinematics

**Fig. 4:**
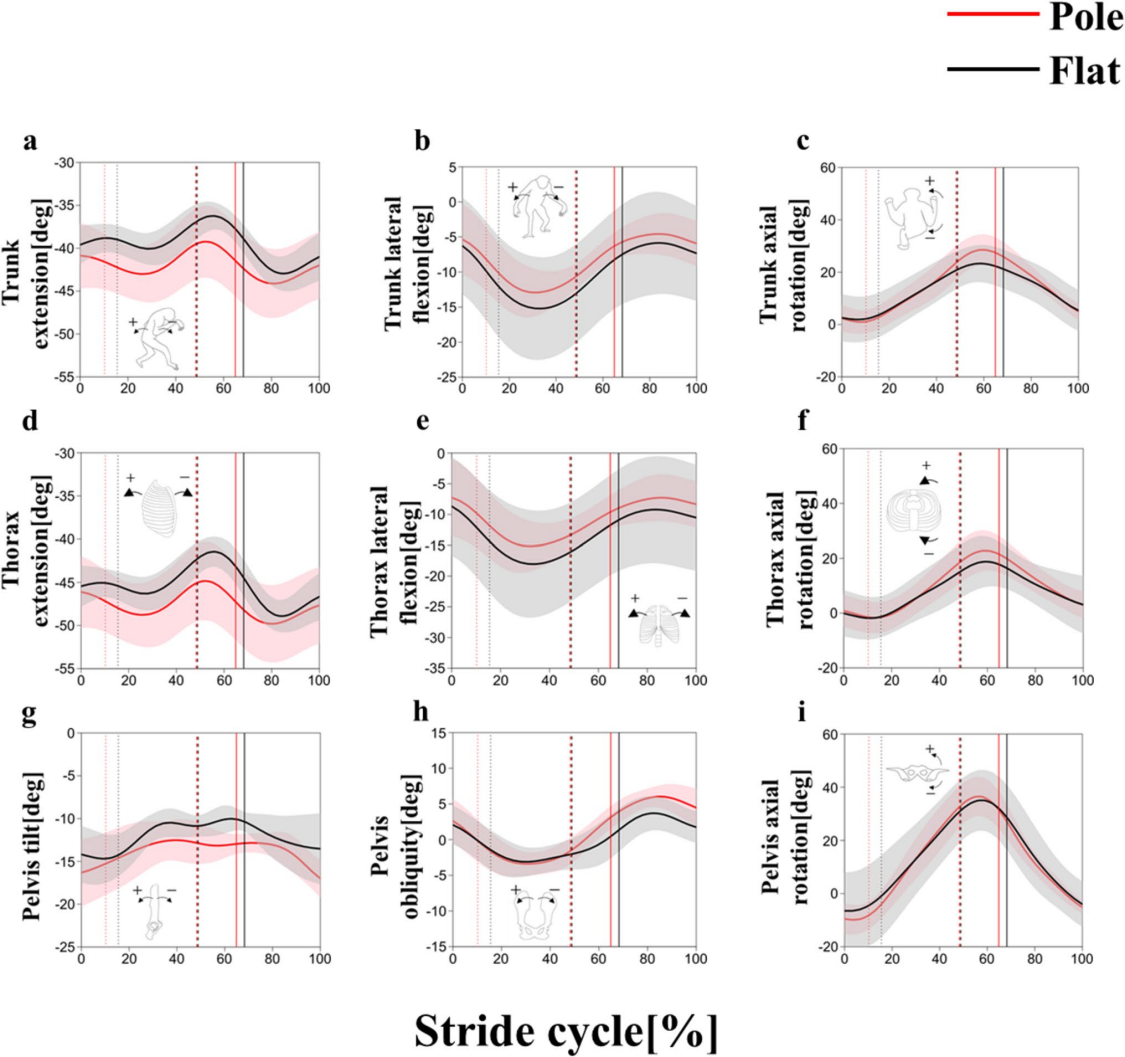
Trunk flexion/extension (**a**), lateral flexion (**b**), axial rotation (**c**), thorax flexion/extension (**d**), lateral flexion (**e**), axial rotation (**f**), pelvis tilt (**g**), obliquity (**h**) and axial rotation (**i**) angles in the left stride cycle between the pole (red) and flat (black) substrate conditions. The thick line represents the mean value, and the shaded area represents the standard deviation ( ±). Each stride cycle begins with a right foot contact and ends on the next right foot contact. The vertical dotted, broken, and solid lines represent the terminal stance of the left foot, its initial contact, and terminal stance of the right foot, respectively

**Fig. 5:**
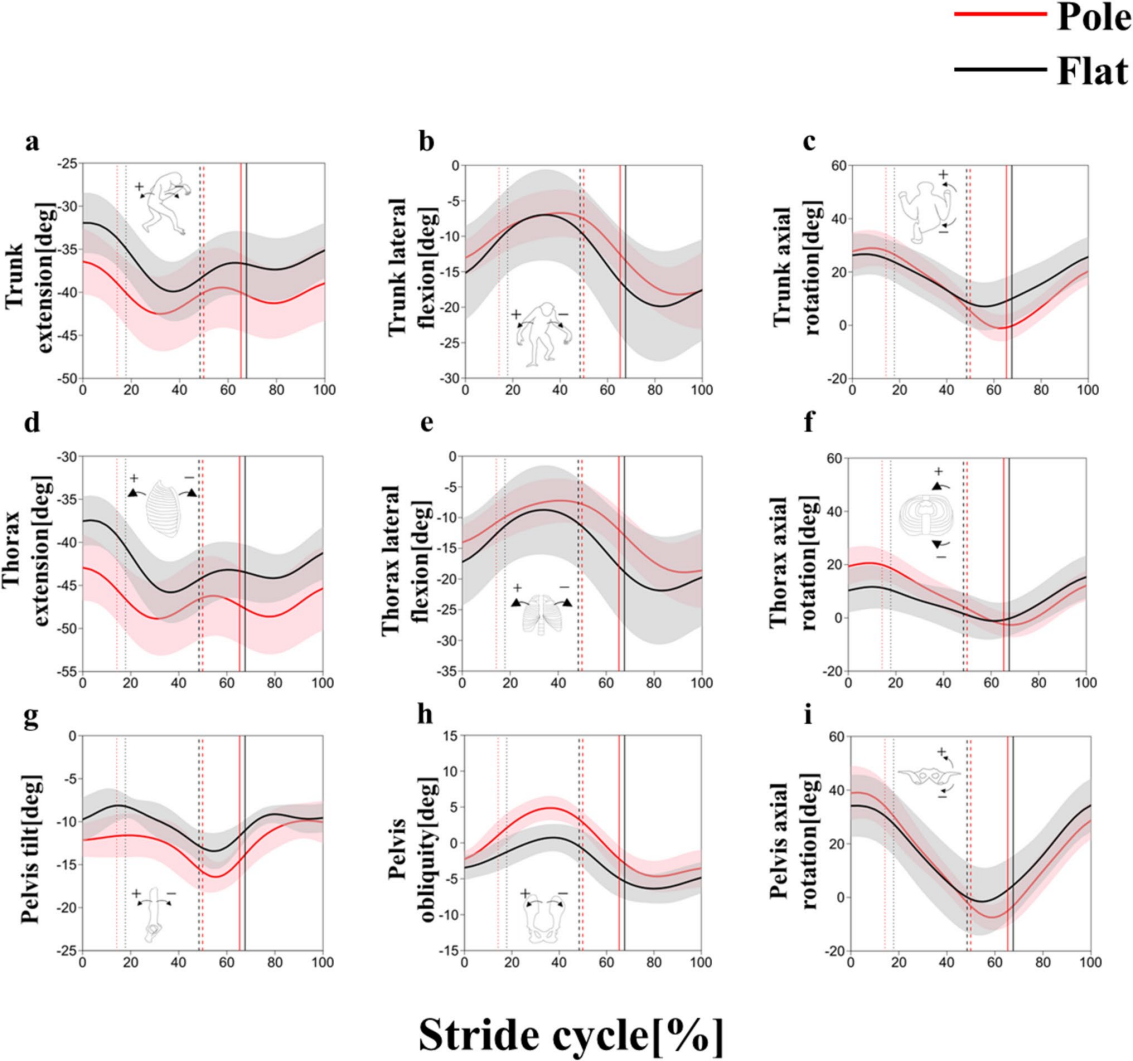
Trunk flexion/extension (**a**), lateral flexion (**b**), axial rotation (**c**), thorax flexion/extension (**d**), lateral flexion (**e**), axial rotation (**f**), pelvis tilt (**g**), obliquity (**h**) and axial rotation (**i**) angles in the right stride cycle between the pole (red) and flat (black) substrate conditions. The thick line represents the mean value, and the shaded area represents the standard deviation ( ±). Each stride cycle begins with a right foot contact and ends on the next right foot contact. The vertical dotted, broken, and solid lines represent the terminal stance of the left foot, its initial contact, and terminal stance of the right foot, respectively

The trunk tended to be more flexed in the pole condition than in the flat condition (Fig. 4a, 5a; Table 3), although statistical significance common to both strides was not obtained during stance phase (Table 3). The statistical significance common to both strides in thorax flexion/extension was not obtained (Figs. 4d, 5d; Table 3). The pelvis exhibited a greater anterior tilt in the pole condition than the flat condition at terminal stance (Figs. 4g, 5g; Table 3).

The statistical significance common to both strides in the trunk and thorax lateral flexion and the pelvis obliquity was not obtained during stance phase (Figs. 4b, e, h, 5b, e, h; Table 3).

The statistical significance common to both strides in the trunk, thorax and pelvis axial rotation was not obtained during stance phase (Figs. 4c, f, i, 5c, f, i; Table 3). The range of motion of trunk rotation was significantly larger in the pole condition than in the flat condition (Table 4).

**Table 4:** Comparison of the amount of the mediolateral center of mass (CoM) displacement and the range of motion of pelvic and trunk rotation during stride cycle between pole and flat conditions

Parameter	Flat	Pole	*p*
The amount of the mediolateral CoM displacement [cm]	$1.9\pm 0.8$	$1.2\pm 0.2$	**
The range of motion of pelvic rotation (right) [deg]	$39.6\pm 4.9$	$47.8\pm 6.8$	*
The range of motion of pelvic rotation (left) [deg]	$45.6\pm 8.7$	$47.8\pm 6.4$	0.70
The range of motion of trunk rotation (right) [deg]	$21.6\pm 3.5$	$30.5\pm 5.0$	**
The range of motion of trunk rotation (left) [deg]	$23.9\pm 4.3$	$31.3\pm 5.7$	**

*p < 0.05, **p < 0.01

Bilateral asymmetries were observed in the trunk and thorax lateral flexion angle (Figs. 4b, e, 5b, e) and the trunk, thorax and pelvis rotation angles (Figs. 4c, f, i, 5c, f, i). The trunk and thorax lateral flexion angle were less than 0° throughout the stride cycle, indicating that the trunk was inclined to the left during walking. Likewise, the trunk, thorax, and pelvis rotation angles were mostly greater than 0 degrees throughout the stride cycle, indicating that the trunk, thorax, and pelvis were facing to the left during walking. These asymmetries were similar in both conditions and may be a characteristic of this individual.

### Hindlimb joint kinematics

**Fig. 6:**
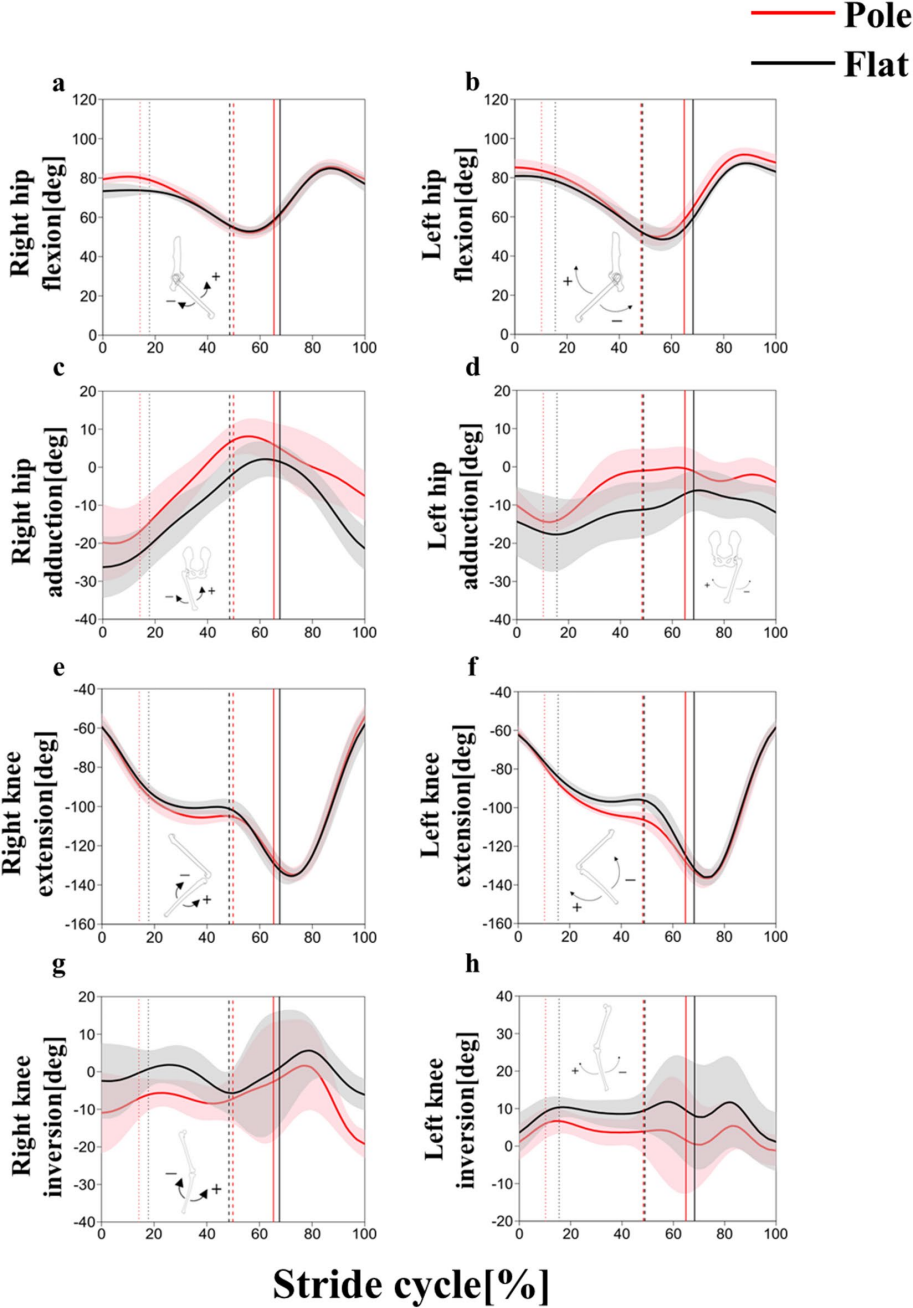
The left and right hip flexion/extension (**a**, **b**), adduction/abduction (**c**, **d**), knee flexion/extension (**e**, **f**) and inversion/eversion (**g**, **h**) angles in the stride cycle between the pole (red) and flat (black) substrate conditions. The thick line represents the mean value, and the shaded area represents the standard deviation ( ±). Each stride cycle begins with a right foot contact and ends on the next right foot contact. The vertical dotted, broken, and solid lines represent the terminal stance of the left foot, its initial contact, and terminal stance of the right foot, respectively

The flexion/extension profiles (Fig. 6a, b, e, f) of both strides were consistent with those reported in previous studies (Vereecke et al. 2006b; Yamazaki and Ishida 1984). In the pole condition, the hip was more flexed than the flat condition at initial contact in both strides (Table 3). The knee was more flexed at midstance in the pole condition than in the flat condition in both strides (Table 3).

Hip abduction/adduction profiles had a similarity and difference between strides (Fig. 6c, d). As a similarity, the hip was abducted from the abduction position at initial contact to the terminal stance, and it was abducted to the next initial contact in both conditions. Hip adduction angle was larger in the pole condition than in the flat condition throughout the stride cycle, with statistical significance at midstance (Table 3). As a difference, the range of motion was smaller in the left stride than in the right stride.

Knee inversion/eversion profiles also had a similarity and difference between strides (Fig. 6g, h). As a similarity, knee inversion/eversion angle showed double-humped profiles with peaks at midstance and swing phase in both conditions. Knee eversion angle was greater in the pole condition than in the flat condition throughout the stride cycle, with statistical significance at midstance (Table 3). As a difference, the absolute angles were smaller in the left stride than in the right stride, meaning that the left knee joint was relatively inverted than the right knee joint.

Bilateral asymmetry was also observed between the hindlimbs for the hip and knee joint angles. However, the trend in changes of the hip and knee joint angles during the transition from a flat surface to a pole substrate were the same for both strides.

### CoM movement

**Fig. 7:**
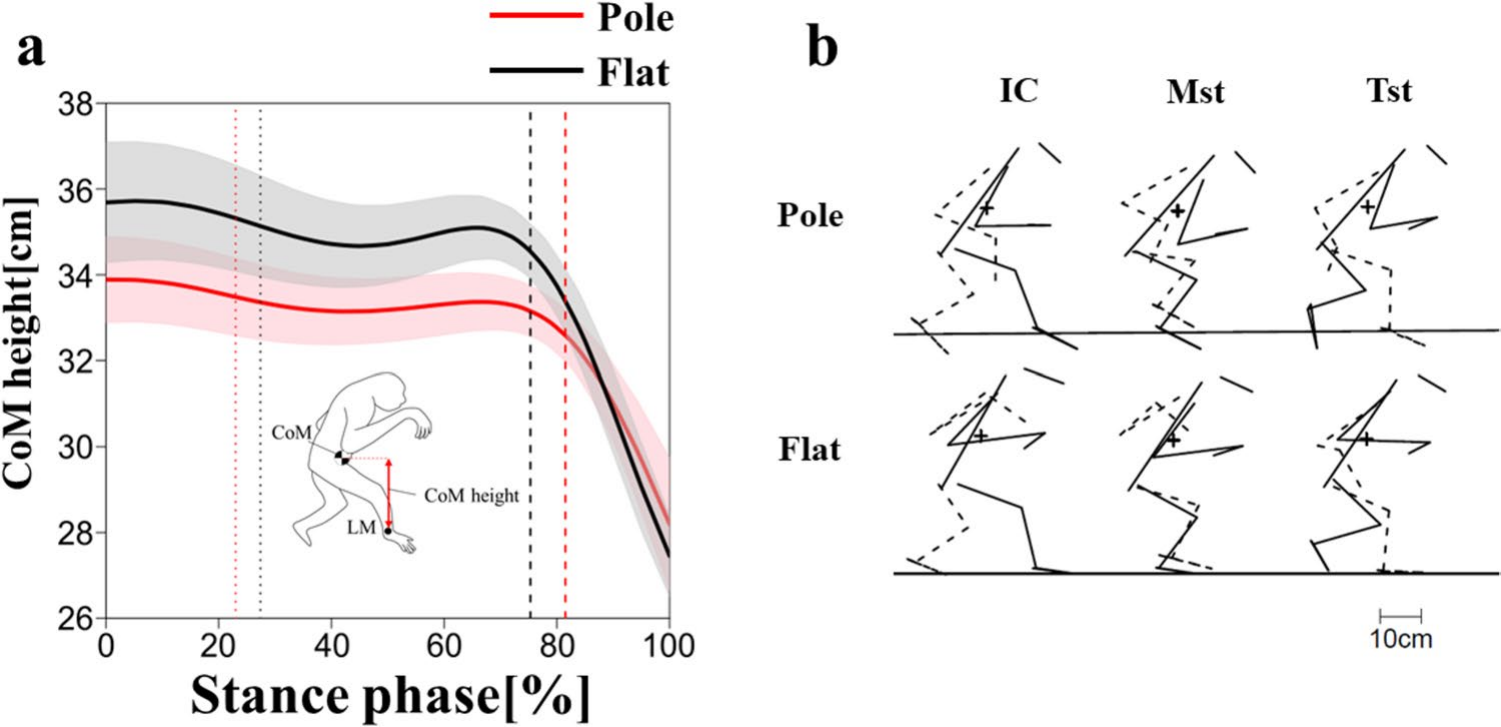
CoM height during stance phase in the pole (red) and flat (black) conditions (**a**). Thick lines represent mean values and shaded areas represent standard deviations ( ±). The vertical dotted and broken lines represent the terminal stance of the left foot and the initial contact of the left foot, respectively. Stick picture of the whole body in the sagittal plane at the initial contact (0% of the stance phase; IC), midstance (50% of the stance phase; Mst), and terminal stance (100% of the stance phase; Tst) (**b**). The trunk segment is represented as a line segment that connected the first thoracic spinous process and the midpoint of both ischial tuberosities. For the limbs, the solid line shows the right side, and the broken line shows the left side. The ‘plus’ signs represent the CoM position. The black horizontal line represents the substrate. Stick picture was plotted as the anteroposterior position of the right calcaneal ridge in both conditions matched in each phase

The CoM height was lower in the pole condition than in the flat condition at initial contact and midstance (Fig. 7a; Table 3). The CoM height was decreased after the initial contact of the contralateral foot (about 80% of the stance phase, Fig. 7a), which was attributed to the elevation of the LM due to ankle plantar flexion at the terminal stance.

**Fig. 8:**
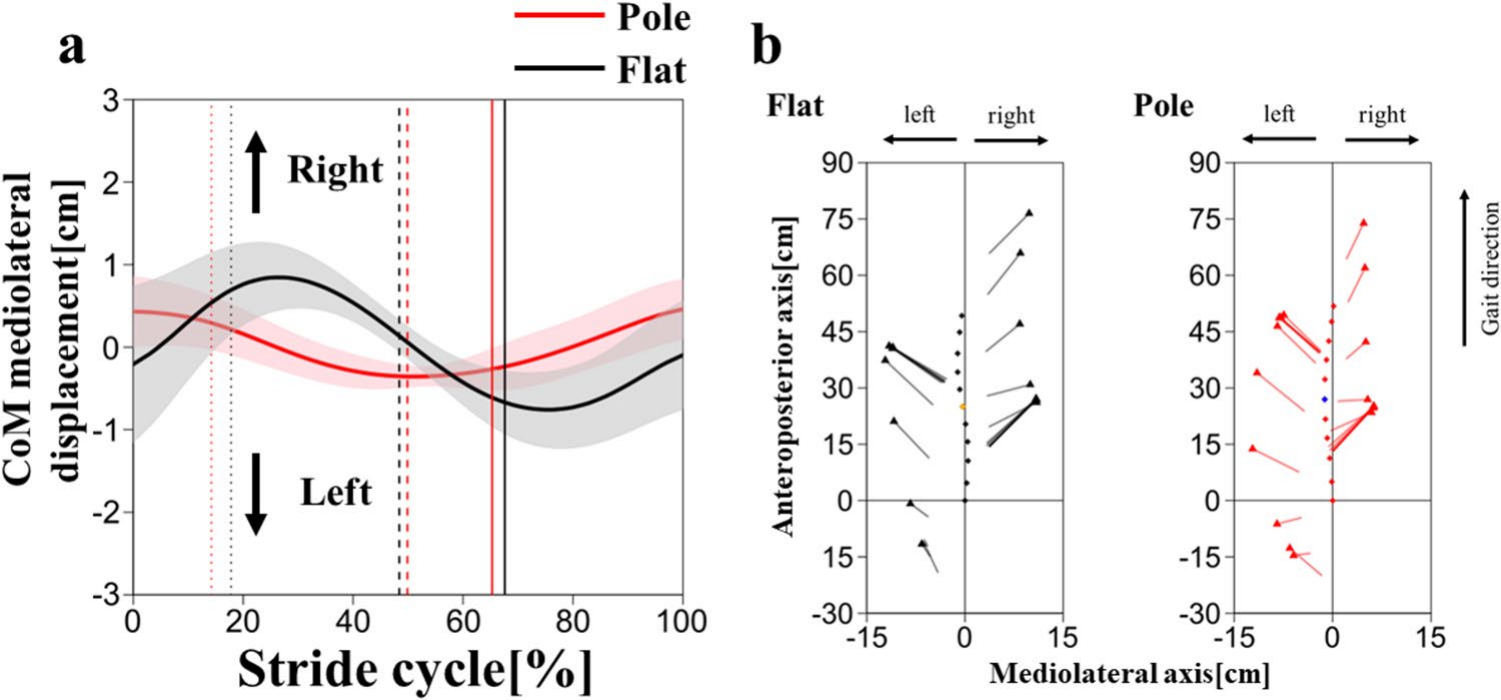
CoM mediolateral displacement on stride cycle during the pole (red) and flat (black) conditions (**a**). Thick lines represent mean values and shaded areas represent standard deviations ( ±). The vertical dotted, broken, and solid lines represent the terminal stance of the left foot, the initial contact of the left foot, and the terminal stance of the right foot, respectively. Relationship between the CoM and both feet in the horizontal plane in the flat and pole conditions (**b**). The filled diamonds indicate the position of the CoM where orange (flat condition) and blue (pole condition) indicate the CoM positions at the initial contact of the left foot. The line segments indicate the feet (connecting the calcaneal ridge and the third foot phalange). The filled triangles indicate the third foot phalange. The gibbon’s initial contact was set as the intersection of the vertical and horizontal lines at zero

**Fig. 9:**
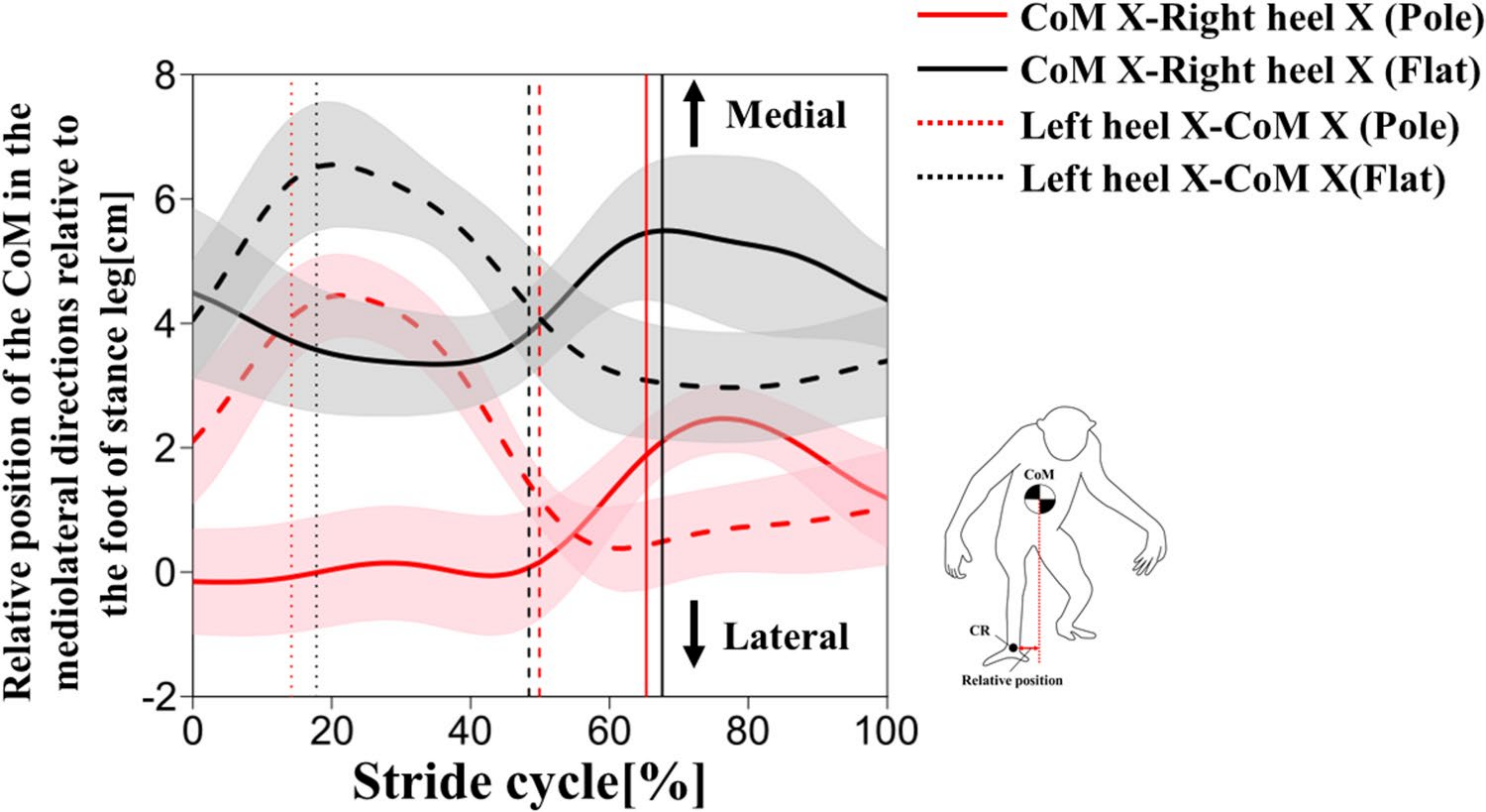
The mediolateral distance between the CoM and the feet (left: broken; right: solid) during stride cycle in the pole (red) and flat (black) conditions. Thick lines represent mean values and shaded areas represent standard deviations ( ±). The vertical dotted, broken, and solid lines represent the terminal stance of the left foot, the initial contact of the left foot, and the terminal stance of the right foot, respectively

The CoM mediolateral displacement showed different trends between conditions (Fig. 8). In the flat condition, the CoM was located at the midpoint of both feet at initial contact, moved to the right side from the initial contact to midstance, moved to the left side from the midstance to the terminal stance, and moved again to the right side from the terminal stance to the next initial contact (Fig. 8). The mediolateral distance between CoM and heel decreased from the initial contact to midstance and increased from midstance to terminal stance in the left and right stance phases (Fig. 9). In the pole condition, however, the CoM was located near the right heel at the initial contact, moved to the left side from the initial contact to the terminal stance, and to the right side from the terminal stance to the next initial contact (Fig. 9). The mediolateral distance between the CoM and the heel remained nearly constant in the left and right stance phases (Fig. 9). The amount of the mediolateral CoM displacement was smaller in the pole condition than in the flat condition (Table 4).

**Fig. 10:**
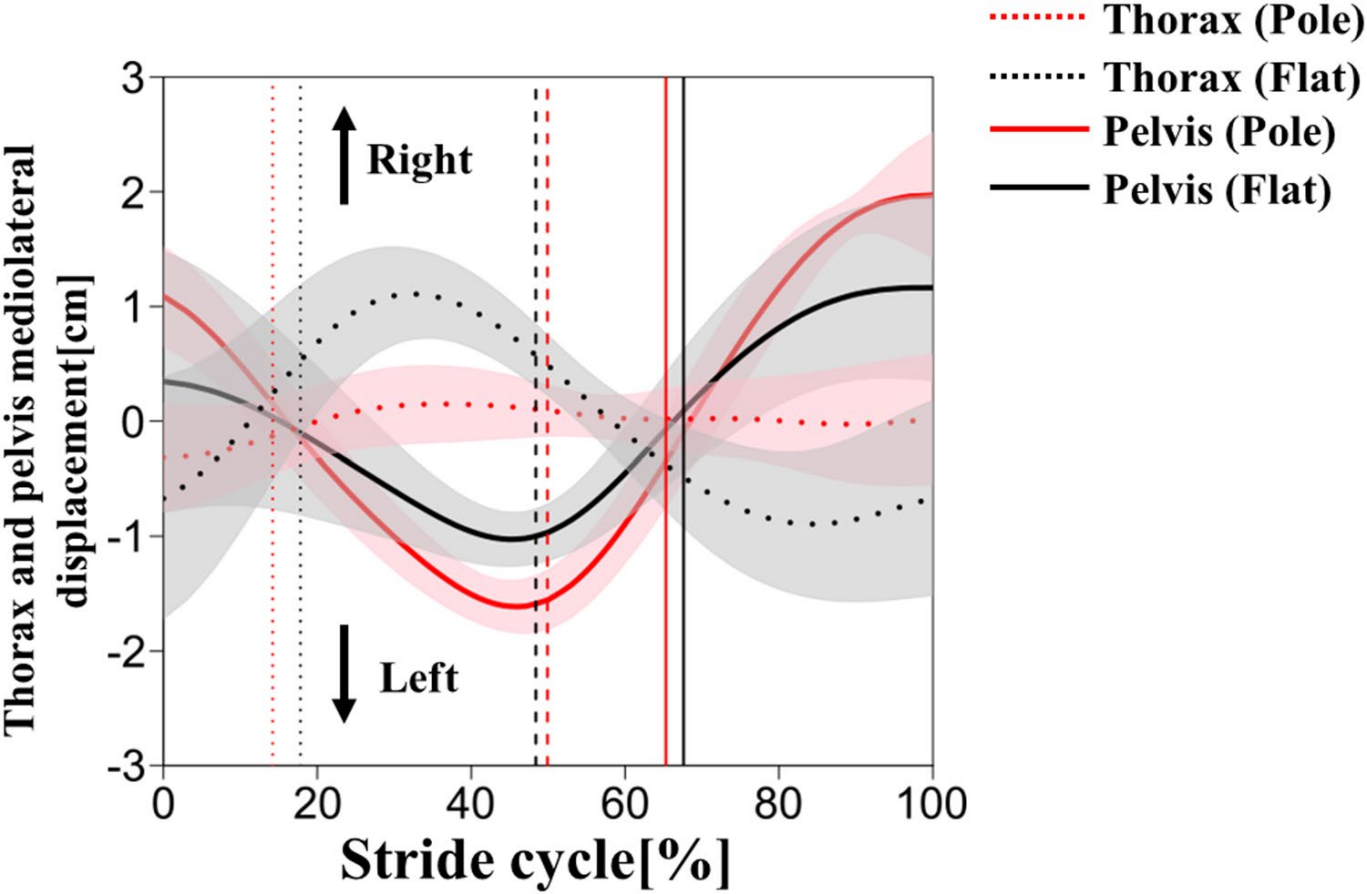
Thorax (dotted) and pelvis (solid) mediolateral displacements during stride cycle in the pole (red) and flat (black) conditions. Thick lines represent mean values and shaded areas represent standard deviations ( ±). The vertical dotted, broken, and solid lines represent the terminal stance of the left foot, the initial contact of the left foot, and the terminal stance of the right foot, respectively

Trunk CoM mediolateral displacement was similar to body CoM mediolateral displacement (Fig. S1), suggesting that trunk motion had an effect. To elucidate the difference of the body CoM mediolateral fluctuation pattern between conditions, the trunk was separated into the thorax and pelvis segments and the mediolateral displacement of each segment was calculated. The pelvis mediolateral displacement showed similar trends between conditions (Fig. 10). The pelvis was displaced to the left from the right initial contact to the left initial contact, and to the right from the left initial contact to the right next initial contact (Fig. 10). The range of motion of the pelvis was greater in the pole condition than in the flat condition (Table 4). On the other hand, the thorax showed different trends between conditions (Fig. 10). In the flat condition, the thorax was displaced to the right from the initial contact to midstance, to the left from midstance to mid swing, and to the right from the next initial contact (Fig. 10). In the pole condition, however, the thorax had less displacement in the mediolateral direction in the stride cycle (Fig. 10).

## Discussion

### Comparison of spatiotemporal parameters between pole and flat conditions

All of the parameters, apart from duty factor in left stride and step width, did not differ between conditions in both strides (Table 2). However, Vereecke et al. (2006a) indicated that gibbons walking on a pole had a relatively larger duty factor and stride length, as well as a relatively lower stride frequency than among those walking on a catwalk (Table 2). In this study, the dimensionless velocity was smaller than that in Vereecke et al. (2006a), which indicates that the gibbon that was used in this study walked at a relatively slow. At the same dimensionless velocity, the duty factor was similar to that reported by Vereecke et al. (2006a), while the dimensionless stride length in this study was relatively small, and the dimensionless stride frequency was relatively large (Table 2). This discrepancy may have been caused by differences in the substrate height. The pole substrate was placed at 100 cm above the ground in Vereecke et al. (2006a), whereas it was placed at 25.6 cm above in this study. The gibbons in the previous study may have moved more quickly due to a fear of falling from the substrate. In addition, the diameter of the pole in this study was smaller than that used in Vereecke et al. (2006a) (approximately 2/3), which may have produced caution in walking (to keep the gibbon from falling off of the substrate), leading to a relatively low average velocity.

### CoM height and joint angles in the sagittal plane during pole walking

The CoM was significantly lower in the pole condition than in the flat condition at initial contact and at midstance (Fig. 7a), indicating that the gibbon in this study kept its body close to the substrate while walking. The gibbon did not simultaneously flex all hindlimb joints but rather flexed the hip and the knee at initial contact and midstance, respectively (Figs. 6a, b, e, f, 7b). Instead, the gibbon flexed the trunk more greatly throughout the stance phase (Figs. 4a, d, 5a, d, 7b). These results indicate that the gibbon may have lowered its CoM primarily by flexing the trunk in the pole condition, and the contribution of the hip and knee joint kinematics in lowering the CoM may be restrictive. It is important to note that, with a lateral sway of the CoM measuring 1 cm and a CoM height of 35 cm, as observed in the present study, the reduction in the moment arm of the gravitational force due to a 2 cm decrease in CoM height is only 0.5 mm. This minimal change suggests that the reduction in CoM height did not significantly contribute to enhanced stability while walking on a cylindrical substrate with a 10 cm diameter.

### The difference of the mediolateral displacement pattern of the CoM between conditions

**Fig. 11:**
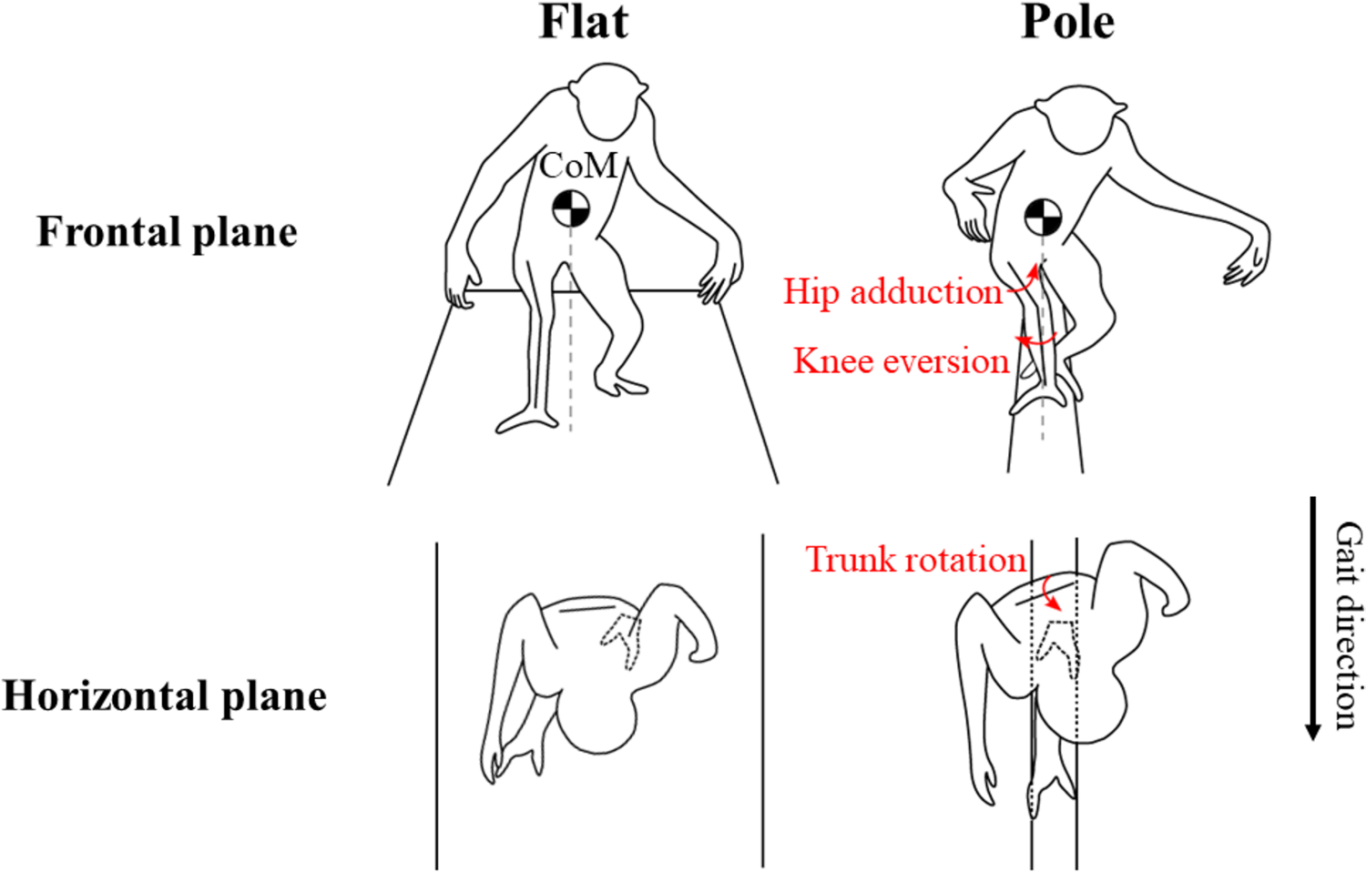
Schematic relationships between the CoM and hindlimb in the frontal plane at midstance (top row) and between the contact point of the left foot and the axial rotation of the trunk in the horizontal plane at the terminal stance (bottom row). The solid lines in the thigh and shank represent the rough placement of the bones, and the dashed lines represent the gravity lines (top row). Note that increased hip adduction and knee eversion in arboreal walking brings the knee and foot closer to the gravity line. The solid horizontal line in the trunk represents the rough line segment that connects both iliac crests (bottom row). Note that the increased right rotation angle of pelvis and trunk in arboreal walking allows the feet to be placed in a straight line

Step width in the pole condition was significantly smaller than that in the flat condition (Table 2), which is caused from the limited foot contact area due to the narrowness of the substrate. We observed that the gibbon increased the angles of hip adduction and knee eversion in the pole condition to reduce the step width in both strides (Fig. 6c, d, g, h). The increased knee eversion angle witnessed in gibbon in pole walking, as their femur is not morphologically inclined like humans’ (Walmsley 1933), allows maintaining the knee joint relatively medially and contributes to their ability to grasp the pole during the single support phase, placing the knee and foot directly below the body (Fig. 11).

A reduced step width may lead to a reduced magnitude for the mediolateral deviation of the CoM in pole walking (Table 4). The gibbon needs to keep the CoM within the base of support to avoid falling to one side. The pole substrate restricts foot placement and compels a narrower step width, resulting in minimal mediolateral deviation of the CoM, thereby keeping it within the narrow base of support.

In the flat condition, the CoM gradually shifted toward the support leg side after the initial contact placed most outwardly at the midstance (Fig. 8a). A similar CoM trajectory has been observed in humans and chimpanzees (Thompson et al. 2018). In bipedal walking, the CoM must be placed above the stance foot to prevent the body from falling to the swinging side during the single support phase. Thompson et al. (2018), who measured the linear mediolateral displacement pattern of the thorax and pelvis in chimpanzees, reported that the thorax was displaced toward the stance side after the foot contact, likely bringing the CoM closer to the stance leg during the single support phase. Similarly, in this study, the mediolateral distance between the CoM and the stance foot gradually decreases after foot contact (Fig. 9), and the thorax was displaced to the stance side during the first half of the stance phase in the flat condition (Fig. 10). These results indicate that the gibbon also shifted the body CoM onto the supporting leg in the flat condition and suggests that the gibbon in this study followed the same strategy as the chimpanzees.

On the other hand, in the pole condition, we observed another trajectory pattern, in which the CoM was shifted to the side of the swinging leg from the initial contact to the midstance (Fig. 8a). The mediolateral distance between the CoM and the stance foot remained constant during stance phase (Fig. 9), which suggests that the foot is already positioned near the CoM at the foot contact. A smaller step width would eliminate the necessity to displace the CoM to the stance side after foot contact, which may cause forward-shifted pattern of the CoM mediolateral displacement (Fig. 9).

The amount of the thorax mediolateral displacement was smaller in the pole condition than in the flat condition (Fig. 10). The gibbon may have restricted the mediolateral motion of the thorax because excessive displacement of the thorax to the stance (lateral) side after foot contact increases the risk of falling. According to the results of flat condition, the changes of the thorax mediolateral motion are similar to that of the CoM mediolateral displacement, suggesting that the thorax movement is closely related to the body CoM movement. In the pole condition, the amount of the pelvis mediolateral motion was larger than that of the thorax, which suggest that the body CoM changes may have been affected by the pelvic segment movement.

The increased range of the trunk rotation (Table 4) in the pole condition could be related to the issue of foot placement, which is unique to the narrow substrate. In the pole condition, the gibbon needs to position its left and right feet so that the line connecting them aligns with the long axis of the pole (Fig. 11). The rotation of the trunk could contribute to bringing the hip joints closer together in the mediolateral direction, resulting in a placement of the left and right feet in a straight line. To achieve the proper foot placement for the pole substrate, postural adjustments may be required of the trunk and lower limb in the frontal plane, as well as in the horizontal plane. In both strides, stride length and right step length were not significantly different between conditions, while left step length was larger in the pole condition than in the flat condition (Table 2). Trunk and pelvic rotation range of motion in the pole condition may have contributed to the increase in left step length (Table 4).

## Limitations

In this study, the detailed 3D kinematic characteristics of a gibbon pole walking was revealed for the first time, but some limitations must be acknowledged. First, the number of individuals is an issue. Although limb movements in the sagittal plane were qualitatively consistent with those reported in previous studies (Vereecke et al. 2006b; Yamazaki and Ishida 1984), apart from the present study, no reports are available concerning 3D limb movements in arboreal environments, and it remains unclear whether similar kinematic adjustments occur in other individuals not studied here. The 3D kinematic data of multiple individuals are necessary to determine this. However, it is quite difficult to access to such data in extant apes (including gibbons), as acquiring it requires anesthesia and shaving. The application of recently developed technologies, such as DeepLabCut (Mathis et al. 2018), that capture body movements without markers (Labuguen et al. 2021; Wiltshire et al. 2023), to gibbons in wild environments or zoos could help in measuring 3D body movements in bipedal walking in a large number of individuals.

Second, the calculated CoM positions may not be entirely accurate due to the method employed to determine the mass percentage of each segment. In this study, the weight of each segment was estimated based on the data provided by Isler et al. (2006). Discrepancies were observed between the measured total body mass and the sum of segment masses derived from external measurements in Isler et al. (2006). Consequently, we recalculated the segment weights, taking account of the impact of removing the viscera. On the other hand, two gibbon specimens (Hy2 and Hy3) from Isler et al. (2006), which exhibited a relatively greater degree of discrepancy, were both fixed in formalin. It has been reported that formalin fixation causes approximately 20% shrinkage in tissue of cadaver specimens (e.g., Su et al. 2006), suggesting that the effect of formalin fixation may be more responsible for the discrepancy than the presence or absence of viscera. Therefore, the method used in this study may have overestimated the weight of the trunk, and a more careful reexamination may be needed for a more accurate CoM position determination. However, there is no doubt that trunk weight represents a relatively large proportion of total body weight, and we believe that the overall trend in CoM behavior observed across different substrates is unlikely to be substantially affected by the variations in the calculation methods.

Third, an additional kinetic study must be performed on pole walking. In this study, hip and knee were flexed to a greater degree at initial contact and midstance in the pole condition than in flat condition (Fig. 5a, b, e, f), which suggests that the gibbon may have adopted a compliant gait, similar to that of other quadrupeds (Schmitt 1999). The functional advantage of a compliant gait is its reduction of the peak of the vertical substrate reaction force component and avoidance of branch vibration (Schmitt 1999). To determine whether a gibbon lowers the peak vertical substrate reaction force component in pole walking, it is necessary to measure the force of the substrate reaction while walking on a pole substrate, as in a previous study (Yamazaki and Ishida 1984). In addition, when gibbons walk on a pole, they generate a torque around the long axis of the substrate, which can be controlled by the frictional forces that are generated between the pole substrate and the plantar surface of the grasping feet. To determine how the frictional force contributes to gibbons’ bipedal walking on a pole, it is necessary to measure the reaction force of the pole substrate while it is being walked upon. The importance of torque around the long axis has recently begun to be more recognized. For example, Lammers and Gauntner (2008) measured torque in quadrupedal walking on a pole in opossums and attempted to separate the torque caused by the position of the CoM and that caused by frictional forces. A mechanical approach similar to this could contribute to the elucidation of mediolateral stability in arboreal bipedal locomotion in gibbons. Furthermore, if CoP could be calculated with the substrate reaction force data, the CoM height could be calculated as the distance from CoP to CoM and compared in both conditions at initial contact, midstance, and terminal stance. In the future, we intend to study how gibbons interact with the environment while walking on unstable pole substrate in more detail and from a kinetic viewpoint.

## Supplementary Information

Below is the link to the electronic supplementary material.

Supplementary file1 (DOCX 83 KB)

## Data Availability

The data supporting the results of this study are available from the corresponding author upon reasonable request.
