# Supplementary material for: A three-dimensional kinematic analysis of bipedal walking in a white-handed gibbon (Hylobates lar) on a horizontal pole and flat surface
Source: Primates. 2025 Jan 15;66(2):189–206. doi: 10.1007/s10329-024-01177-x (PMC11861135; doi:10.1007/s10329-024-01177-x)
Supplement: Supplementary file 1 — Supplementary file1 (DOCX 83 KB) [file 10329_2024_1177_MOESM1_ESM.docx]

**Supplementary Information: A three-dimensional kinematic analysis of bipedal walking in a white-handed gibbon (*Hylobates lar*) on a horizontal pole and flat surface**

Takafumi Fujiwara^1^・Kohta Ito^1, 2^・Tetsuya Shitara^1^・Yoshihiko Nakano^1^

^1^Laboratory of Biological Anthropology, Graduate School of Human Sciences, Osaka University, Suita, Osaka, Japan

^2^Artificial Intelligence Research Center, National Institute of Advanced Industrial Science and Technology (AIST), Koto-ku, Tokyo, Japan

Tel: +81-6-6879-8057

E-mail: [t.fujiwara.osakauniv@gmail.com](mailto:t.fujiwara.osakauniv@gmail.com)

Detailed explanation of the definition of the local coordinate system for the pelvis,　trunk, thigh, and shank segments

The thorax coordinate system:

First, the z axis was defined as a vector from the thirteenth thoracic spinous process (T13) to the first thoracic spinous process (T1); then, the temporal y axis was defined as a vector from T1 to the midpoint of the nipples (NI); the x axis was calculated as the outer product of the y and temporal z axes; and finally, the y axis was calculated as the outer product of the z and x axes.

The pelvic coordinate system:

First, the z axis was defined as a vector from the midpoint of the ischial tuberosities (IT) to the midpoint of the iliac crests (IC), and the temporal x axis was defined as a vector from the midpoint of the ischial tuberosities to the right ischial tuberosity; then, the y axis was calculated by the outer product of the z and temporal x axes, and finally, the x axis was calculated by the outer product of the y and z axes.

The trunk coordinate system:

First, the z axis was defined as a vector from the midpoint of the iliac crests (IC) to the first thoracic spinous process (T1), and the temporal x axis was defined as a vector from the midpoint of the iliac crests to the right iliac crest; then, the y axis was calculated by the outer product of the z and temporal x axes, and finally, the x axis was calculated by the outer product of the y and z axes.

The right thigh coordinate system:

First, the z axis was defined as a vector from the femoral lateral epicondyle (LE) to the greater trochanter (GT); then, the temporal x axis was defined as a vector from the femoral medial epicondyle (ME) to the femoral lateral epicondyle (LE); the y axis was calculated as the outer product of the z and temporal x axes; and finally, the x axis was calculated as the outer product of the y and z axes.

The left thigh coordinate system:

First, the z axis was defined as a vector from the femoral lateral epicondyle (LE) to the greater trochanter (GT); then, the temporal x axis was defined as a vector from the femoral lateral epicondyle (LE) to the femoral medial epicondyle (ME); the y axis was calculated as the outer product of the z and temporal x axes; and finally, the x axis was calculated as the outer product of the y and z axes.

The right shank coordinate system:

First, the z axis was defined as the vector from the lateral malleolus (LM) to the tibial lateral condyle (LC); then, the temporal x axis was defined as the vector from the medial malleolus (MM) to lateral malleolus (LM); then, the y axis was calculated as the outer product of the z and temporal x axes; and finally, the x axis was calculated as the outer product of the y and z axes.

The left shank coordinate system:

First, the z axis was defined as the vector from the lateral malleolus (LM) to the tibial lateral condyle (LC); then, the temporal x axis was defined as the vector from the lateral malleolus (LM) to medial malleolus (MM); then, the y axis was calculated as the outer product of the z and temporal x axes; and finally, the x axis was calculated as the outer product of the y and z axes.

　　　
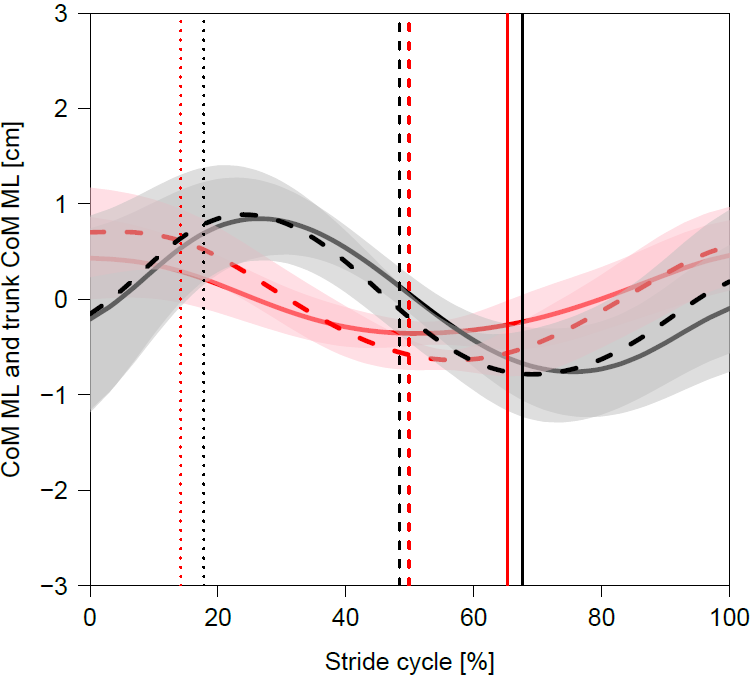


Fig. S1 Body (solid) and trunk (broken) CoM mediolateral displacement on stride cycle during the pole (red) and flat (black) conditions. Thick lines represent mean values and shaded areas represent standard deviations (±). The vertical dotted, broken, and solid lines represent the terminal stance of the left foot, the initial contact of the left foot, and the terminal stance of the right foot, respectively. Note the similarity of the body and trunk CoM profiles in both conditions.

Table S1 The mass, mass percentage, and CoM position (as a percentage of segment length from the proximal point) for each segment of Hylobates lar (Isler et al., 2006). The mass of the trunk segment was recalculated by subtracting the total mass of the non-trunk segments from the body weight (BW) to estimate the value including viscera. Note that the mass of the trunk segment does not match the value reported in Table 3 of Isler et al. (2006), as that measurement was taken after evisceration.

|  | Head | Trunk | Upper arm | Forearm | Hand | Thigh | Shank | Foot |
| --- | --- | --- | --- | --- | --- | --- | --- | --- |
| Mass of segment [kg] |  |  |  |  |  |  |  |  |
| Hy1 (BW: 4.65kg) | 0.376 | 2.648 | 0.219 | 0.143 | 0.044 | 0.256 | 0.104 | 0.047 |
| Hy2 (BW: 5.4kg) | 0.383 | 3.947 | 0.118 | 0.112 | 0.045 | 0.130 | 0.077 | 0.053 |
| Hy3 (BW: 6.9kg) | 0.433 | 4.665 | 0.231 | 0.186 | 0.088 | 0.223 | 0.105 | 0.068 |
| Mass percentage [%] |  |  |  |  |  |  |  |  |
| Hy1 | 8.09 | 56.94 | 4.71 | 3.08 | 0.95 | 5.51 | 2.24 | 1.01 |
| Hy2 | 7.09 | 73.09 | 2.19 | 2.07 | 0.83 | 2.41 | 1.43 | 0.98 |
| Hy3 | 6.28 | 67.61 | 3.35 | 2.70 | 1.28 | 3.23 | 1.52 | 1.00 |
| CoM position percentage [%] |  |  |  |  |  |  |  |  |
| Hy1 | 43.7 | 48.2 | 46.2 | 42.9 | 49.5 | 44.0 | 45.7 | 49.6 |
| Hy2 | 45.9 | 43.0 | 50.0 | 44.1 | 52.0 | 50.0 | 50.6 | 47.5 |
| Hy3 | 43.9 | 49.4 | 48.0 | 46.1 | 50.3 | 43.7 | 44.4 | 48.4 |
